# Planning and delivering co-creation workshops: practical lessons from digital health device design

**DOI:** 10.3389/fdgth.2026.1764878

**Published:** 2026-03-17

**Authors:** Aidan McConnell-Trevillion, Lynda Webb, Wei Ju, Srinjoy Mitra, Lynne Baillie, Kianoush Nazarpour

**Affiliations:** 1 School of Informatics, The University of Edinburgh, Edinburgh, United Kingdom; 2 School of Engineering, The University of Edinburgh, Edinburgh, United Kingdom; 3 School of Mathematical & Computer Sciences, Heriot Watt University, Edinburgh, United Kingdom

**Keywords:** biomedical device development, co-creation, digital health, overactive bladder, participatory design, stakeholder engagement

## Abstract

Co-creation methods are increasingly recognised as essential in digital health and care, yet engineers and physical scientists new to the field often find the literature highly theoretical, fragmented, and difficult to apply in practice. This paper presents a worked example of planning and delivering co-creation workshops through the development of an overactive bladder treatment device. Drawing on established participatory design frameworks, we situate our approach within existing theory while providing a modular toolkit structured around purpose (Why), content (What), and delivery (How). The toolkit is not intended as a new theoretical contribution, but as an accessible starting point for engineers and researchers seeking to integrate stakeholder engagement into biomedical device development. We also describe the use of an electronic whiteboard environment for rapid data capture and organization, reducing the need for full transcription and supporting efficient translation of stakeholder input into actionable design insights. By illustrating the process end-to-end and aligning it with key principles from the co-design literature, this paper provides early-career researchers and engineers with a concise, practice-oriented reference for running effective co-creation activities in digital health and care contexts.

## Introduction

At the turn of the millennium, co-creation was primarily applied in the fields of business and finance (1). However, in the last decade, its use has expanded rapidly into health and care research, building on long-standing traditions of participatory and co-design methods (2–5). Despite this growth, there is still no single, agreed-upon definition or universally accepted methodology for co-creation. To this end, for the purpose of allowing early career researchers, or those inexperienced with co-creation to follow the present methodology, a relatively simplistic definition was taken. Drawing upon multiple sources (6–8), we define co-creation as “a process in which researchers and stakeholders collaboratively contribute to the ideation, planning, implementation, and evaluation of new services and systems, as a means of optimizing the impact of research findings.”

While numerous toolkits and frameworks have been developed to support participatory design in healthcare settings (4, 5), and more recently within quantitative fields such as computer science (9), variation in how co-creation projects are structured, analysed, and reported persists (10, 11). This variation stems both from the lack of a unified definition and from the need to tailor methods to specific stakeholder groups and project goals. Best practice and culture have a long history. The legacy of comparative work in the field of social theory can be traced back at least to the Greek Antiquity and, never interrupted, this sustained tradition has since then been only reinforced as the time has passed. In our own time, due to certain historical developments like the enormous increase in communications, technological advances and the immanent intensification of internationalisation tendencies, comparative research, especially cross-national comparison, has increasingly been receiving much attention. As a result, the bulk of contemporary human and social sciences abounds with examples of comparative approaches. In this section we shall just take a brief look at a rather arbitrarily chosen set of studies, belonging to different fields, in which this methodological strategy has been adopted in order to demonstrate the spread and general applicability of early stakeholder involvement in research across disciplines (7, 12). Many studies prioritise solving narrowly defined problems rather than seeking deeper *insight* into stakeholders’ lived experiences and broader systemic factors such as manufacturing or service delivery.

We define insight as a deep understanding of how the target population experiences a problem or condition and their personal relationship to it or to potential solutions. This differs from context, which can often be inferred through observation or literature review alone. Capturing insight requires direct and meaningful engagement with stakeholders. Without it, co-creation risks missing unmet needs and failing to generate truly user-centred innovations, an issue highlighted repeatedly in participatory design literature (4, 5).

Another recurring challenge is the capture, synthesis, and dissemination of data generated during co-creation. Traditional methods rely on extensive audio/video transcription or handwritten notes, which can delay projects and dilute findings (13). Moreover, co-creation activities are often dominated by researchers or managerial staff (12), with engineers and technical contributors engaged at a later stage. This tendency can inadvertently reinforce disciplinary silos and limit feasibility-aware insights, despite concerns voiced in design practice communities that early engineering involvement usually saves everyone a lot of time and waste of resources. Although the increasing use of fictitious users (“personas”) to guide engineering practices has brought the idea of co-creation based techniques into the technical space (14, 15), this disciplinary gap has nevertheless led to calls for integrating co-design practices more explicitly into engineering education and biomedical research workflows (16).

This paper does not seek to propose a new theoretical framework or claim methodological novelty. Many of the principles described in this guideline are based upon standard participatory design principles which are expanded upon considerably within the literature (17, 18). However, as has likely been experienced by prospective researchers, these principles are often abstract or lacking in concrete guidance. To this end, the authors produced this document as a succinct worked example and practical guide for engineers, physical scientists, and early-career researchers who are new to co-creation. We present a modular toolkit for planning and delivering co-creation workshops, developed and demonstrated through a case study on wearable treatment devices for Overactive Bladder (OAB). By situating our approach within established participatory design literature (4, 9, 17, 19), we aim to bridge theory and practice, providing a clear and accessible starting point for those wishing to integrate stakeholder engagement into digital health and care innovation.

## Our approach

### The co-creation toolkit

Our toolkit followed the guidance laid out by the “double diamond” design framework (20). A critical aspect of approach taken in the present work was the contextualisation of the condition by allowing the very early involvement of stakeholders in the research process. This contextualisation is an explicit requirement of the present work—one that has been repeatedly highlighted as vital within the healthcare literature space (21–23).

As such, it was considered at every phase of the co-creation planning process. Prospective researchers should be aware that to adequately integrate a concept as broad as “context” into the methodological approach, it must be understood that every research situation has a unique context. Any approach taken should be tailored to maximise the impact of the co-creation study.

The first stage of the planning process was the design of a modular framework comprising three core features: “WHY,” “WHAT,” and “HOW,” each encompassing various subcategories. These features correspond to the timing of the co-creation study within the project (WHY), the overall goal of the intervention (WHAT), and the method by which this goal will be explored or achieved (HOW). A framework based approach to co-creation such as this is not a new concept (4, 19)—often a structured framework such as this is seen in the form of a topic guide used before a semi-structured interview (24, 25). However, it was hoped that by clearly delineating each phase of the project and methodologies employed therein, prospective researchers may easily reproduce or tailor these guidelines to the requirements of their specific projects. To this end, when planning a co-creation workshop, researchers should feel free to combine, modify, or add to the elements of the framework described here to address specific research questions or goals. Any co-creation effort should ideally draw on all three features to develop a tailored, research-specific co-creation framework.

**Figure 1:**
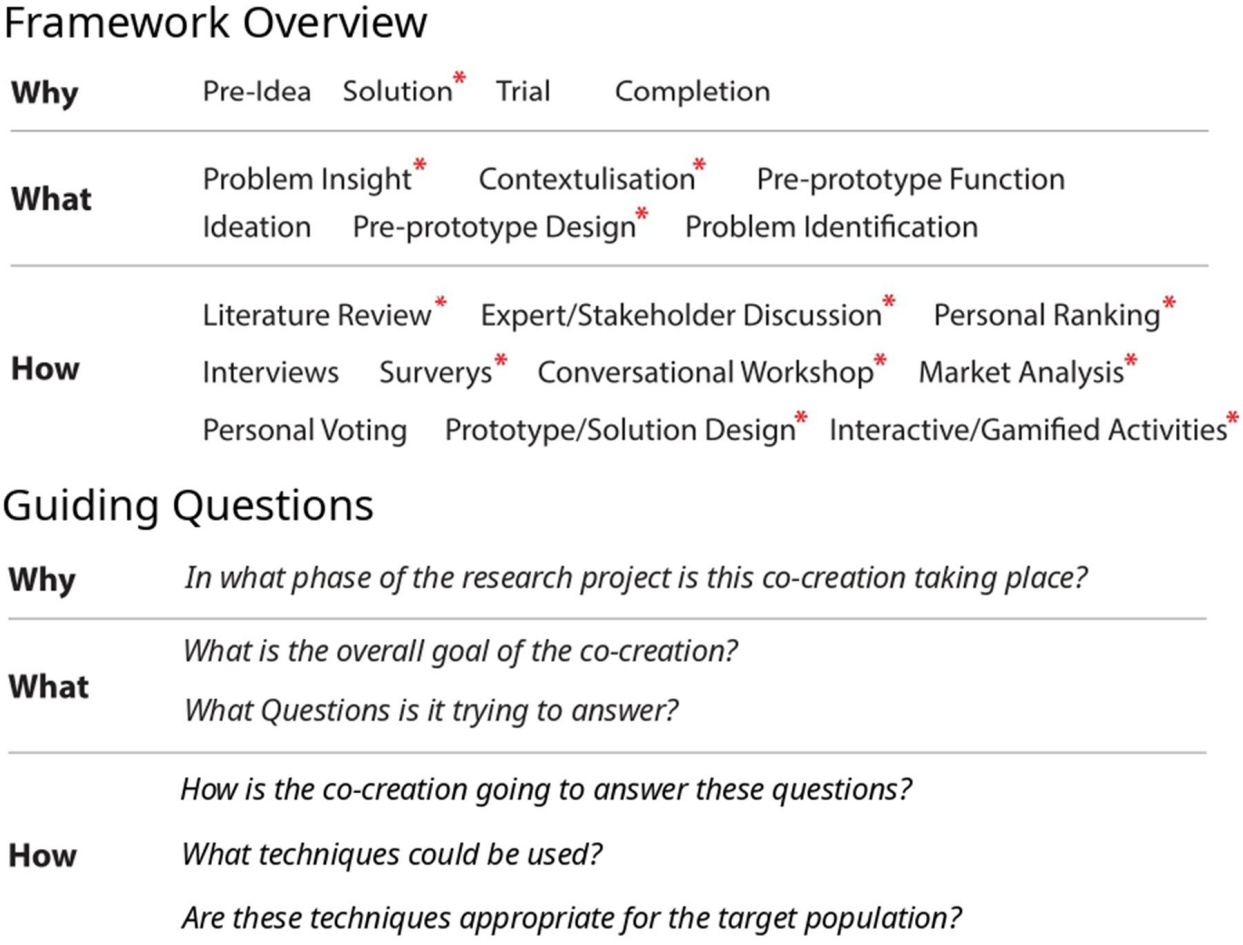
Working toolkit table. Shown are the three factors included in our co-creation framework and their respective features (TOP), and guiding questions for prospective researchers (BOTTOM). Any co-creation project may include any combination of items within each factor. Red asterisks highlight aspects of the toolkit that were used as part of the conducted co-creation case study.

Prospective researchers should note that any project will likely require conducting multiple workshops, each employing different aspects of the toolkit to provide a holistic understanding of a problem space. As such, researchers should aim to consider the framework not as a set of separate methodological tools but rather a connected set of features that inform one another. For example, the stage of the research and the motivation for the co-creation (WHY) will influence the objectives of the workshop (the WHAT) and the methods for achieving them (the HOW). Likewise, decisions about goals and techniques will inform the placement of co-creation activities within the broader research timeline. Therefore, to achieve the best possible combination of toolkit resources, it is essential to view the toolkit as a whole, capable of offering a customized approach to specific challenges. To aid in deciding on the specifics of each aspect of the framework, a set of guiding questions have been included (see Figure 1) however, these are by no means an exhaustive list and researchers should feel free to add their own project-specific considerations.

#### WHY we conduct co-creation

The ideal co-creation process should involve stakeholders at every stage of the research. Achieving this requires a clear understanding of the purpose behind each co-creation session—why the session is being held and why stakeholder involvement is necessary. However, this is not a concrete requirement. Where there is very little understanding of the benefits that may be generated by a co-creation workshop, there additionally exist a suite of tools to explore the “unknown unknowns” within the research space. These can include open pilot interviews, or initial focus groups dedicated to exploring the value of prospective research directions—particularly in the fields of engineering (26) and healthcare (27, 28). To this end, prospective researchers should be aware that co-creation can, and should, be readily applied to any stage of a research process.

Co-creation can occur during the initial “pre-idea” phase, where the research team analyzes the current state of the field to identify key questions or unmet needs that would benefit from stakeholder input. Alternatively, it can take place in the “solutions” phase, where potential solutions that meet stakeholders’ needs are developed. Further along, co-creation may be integrated into the “trial” stage, where proposed solutions are tested with stakeholder guidance, or during the “end” phase, where results are contextualised, refined, or wrapped up after trials (see Figure 1).

#### WHAT we wish the co-creation to do

The second feature of the methodological framework defines the overall goal of a specific co-creation endeavor. These goals will vary based on the timing of the workshop within the research project (the WHY) and the tasks most suitable for the stakeholder population (the HOW). Generally, researchers should aim to lay out the specific goals at the specific stage of their research project, such as ideation or problem identification—highlighting specific needs or issues within the stakeholder group; contextualisation of the condition or problem—understanding how stakeholders’ needs manifest in their lives; or more concrete objectives like pre-prototype design and function—such as determining where a device would be placed in a home. The stated goal of the workshop/co-creation endeavor can also involve refining developed devices or strategies, for instance, presenting early designs to stakeholders and gathering iterative feedback (see Figure 1).

Researchers should be aware that the open-ended nature of a co-creation methodology allows for one or more of these goals to be addressed within a single co-creation session. As such, while multiple workshops are likely to be needed throughout the research process due to the iterative nature of co-creation researchers should not become overly constrained in their overall scope/goals.

#### HOW we conduct co-creation

The final feature of the toolkit details the specific techniques and activities that can be used by researchers to achieve their stated co-creation goals (the WHAT), based on the stage of the research project (the WHY). These approaches can range from discovery-oriented techniques, such as preliminary literature reviews, market analyses, expert consultations, or initial stakeholder outreach, to data-generating methods like: interviews, conversational workshops, and interactive activities, including voting, surveys, and gamified strategies. When more concrete information is needed, such as feedback on prototypes, techniques like: brainstorming, solution design, and discussions around example devices or solutions can be particularly useful (see Figure 1).

When designing a co-creation study using the HOW feature, it is recommended to incorporate both open discussions and interactive components like surveys within the same session. This ensures participants can freely express their opinions or record their thoughts without concerns about group disagreement or bias. Moreover, while the present framework provides reproducible guidance - prospective researchers should be open to the prospect of integrating other methodologies if they believe them to be relevant or useful.

Finally, prospective researcher should also consider the setting (or spatial/situational context) of the workshop when deciding upon methodological techniques. The set and setting of a co-creation workshop is critical for participant recruitment, and empowerment (29, 30). In terms of contextualisation, prospective researchers should be open to the idea of conducting workshops under different conditions, to gather a holistic and context-informed overview of participants responses and thoughts. For example, in the context of bladder dysfunction (the focus of the case study described later) conducting co-creation workshops in a public (e.g., research labs or clinics) and domestic (e.g., participant’s homes) settings can provide critical context to participant responses, potentially highlighting further “unknown-unknowns” that might not have been clear otherwise. Regardless of the setting utilised however, researchers should make every effort to ensure that participants feel safe, and empowered by their taking part in the work (31).

### Means of recording

Another critical consideration made by researchers during the process of planning a co-creation event is the means of recording the data generated during the workshop. There are an extensive and varied set of methodologies within the qualitative research space, both generally applicable and specialised dependent on use-cases. As such, prospective researchers should decide an appropriate technique based on their research requirements (e.g., the type of data gathered, tools employed etc.), available resources, planned analyses (e.g., software suites—data exporting, etc.) and personal preferences.

To provide some guidance for prospective researchers, our approach included the development and use of an electronic data collection method. As previously mentioned, data from co-creation is typically captured via audio or video recording which, is subsequently transcribed for further analysis (13). In most cases, these traditional approaches are more than adequate—though they require that researchers undertake lengthy post-workshop transcription of the qualitative data before analysis. We here describe an alternative method of data collection using freely available digital tools that may prove useful for researchers performing co-creation for the first time. This approach utilises both typical video/audio recording to support the primary means of data collection: an electronic whiteboard environment [Mural (32)].

The use of such electronic (or physical) whiteboards is an established technique within the healthcare co-creation literature and has been used to great effect in the past (33, 34). Its established efficacy and ease of use make it well suited for use by first-time co-creation researchers to produce detailed and transparent results. As will be shown, both data recording and analysis may be performed easily, within the same space—and any results quickly shared between researchers, facilitating the rapid dissemination of results within research groups.

The online environment is able to capture any technique from the HOW toolkit feature and conduct post-study qualitative analysis in a single shared electronic space.

#### The whiteboard environment

**Figure 2:**
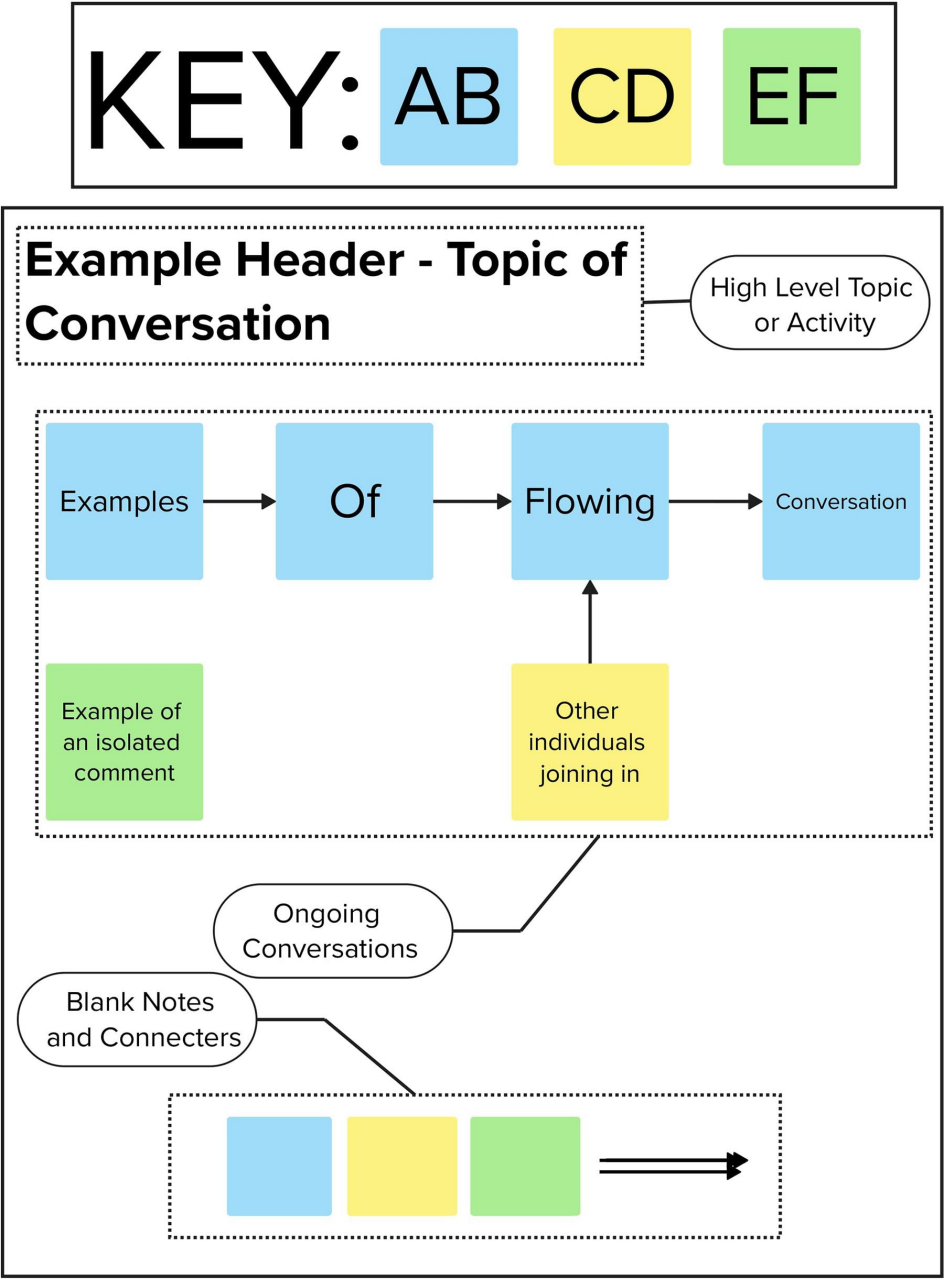
Example section of the electronic whiteboard environment showing: the colored post-it note key (with example initials), high-level conversation topic, examples of flowing conversation between multiple participants and isolated comments, and the reserve of blank notes and connectors for notetakers to allow rapid transcription.

**Figure 3:**
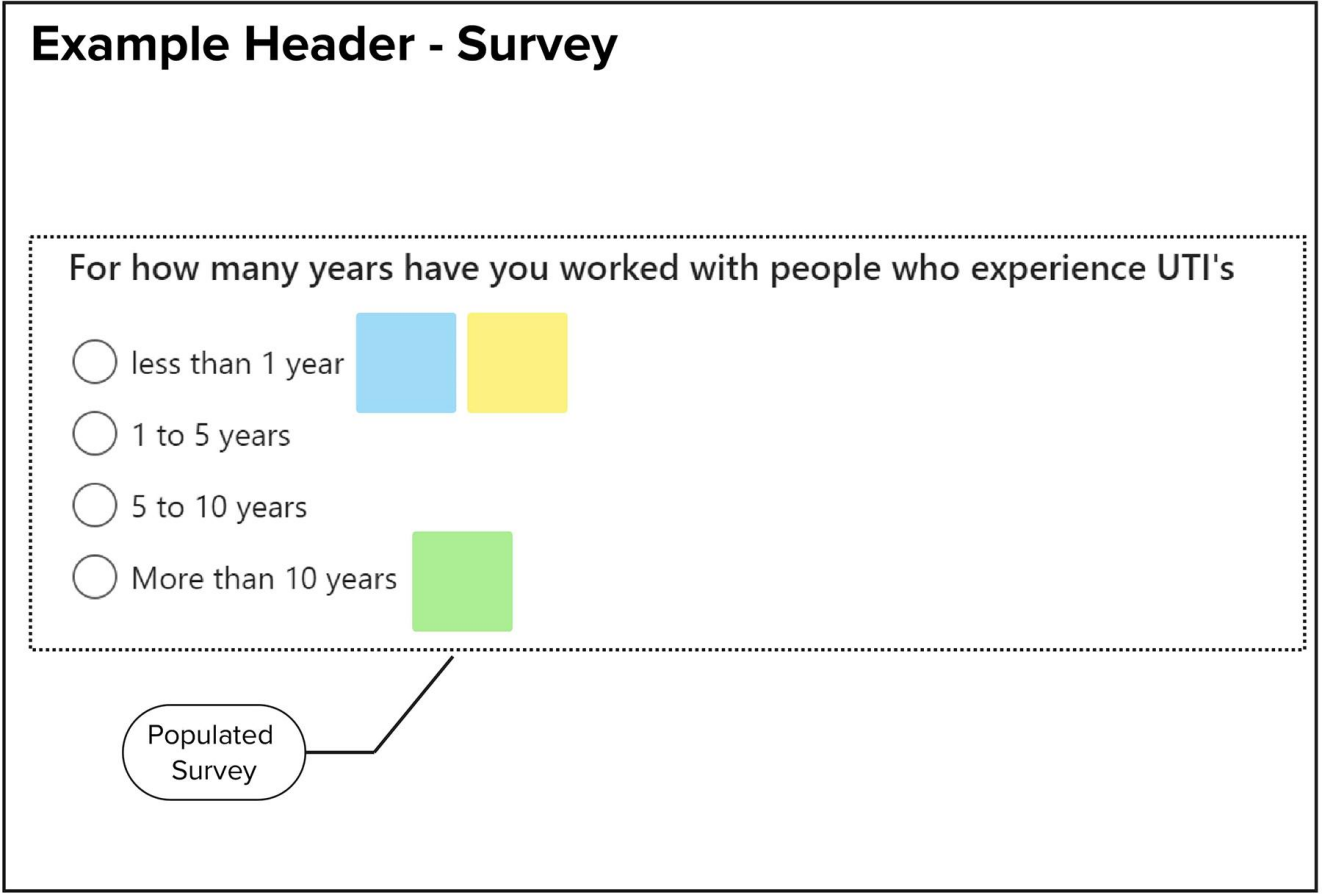
Example of a survey rapidly populated with data within the whiteboard environment.

Each co-creation study is assigned a unique online whiteboard environment, accessible to all researchers involved in the project simultaneously. During the session, high-level topics of discussion or activities—guided by the WHAT and HOW features—are represented as headers on the whiteboard. Participants are each assigned a color-coded “post-it” note within the program, allowing for easy identification. The data generated by each participants is recorded on these notes in real time, either as a line-by-line transcript (see Figure 2) or as responses in a poll or survey (see Figure 3), with each block representing a single sentence, topic, or vote.

Before any co-creation workshop begins, research team members attending as notetakers are each assigned one or two participants to transcribe. The colors assigned to participants are displayed in a key at the top of the whiteboard environment (see Figure 2 top). Notetakers transcribe comments made by their assigned participants using color-coded notes and connect related points with arrows. Where possible, engineers on the project also act as notetakers, helping bridge communication between stakeholders and technical teams, fostering a deeper understanding between social scientists and designers/engineers.

This method effectively distills complex conversations into a clear, structured format. If a participant is not actively contributing, notetakers can assist others, allowing for collaborative error checking during the session. If a notetaker misses any information, they can insert timestamps linked to the ongoing recording, enabling post-workshop corrections.

#### Post-workshop analysis

After the workshop, researchers review the video or audio recording to correct errors and compare it with the whiteboard notes to fill in any gaps, such as timestamps left by notetakers. Once corrections are made, the data is anonymised by deleting the color key, leaving behind a conversation log free of any identifying information.

As mentioned previously, a benefit of using this electronic data collection method is the ability to conduct qualitative analysis of the co-creation data within the same space. Prospective researchers should be aware that there are once again a varied and considerable number of approaches that may be used to analyse qualitative data. We here describe one possible technique—Grounded Theory—however prospective researchers should be aware that a different method of analysis may be more appropriate. Where an analysis cannot be conducted directly upon the Mural space—it is possible to export datasets from the tool where needed.

Fortunately, grounded theory analysis can be carried out directly upon the raw data within the whiteboard environment without the need to export the data. Moreover, for most use-cases it serves as an appropriate method of analysis. One subtype of grounded theory analysis is particularly useful in the analysis of relatively open-ended co-creation data—the constant comparative method (CCM). This approach allows themes to emerge from the dataset without bias (35, 36) and may be readily applied to Mural data using the inbuilt tagging system—though prospective researchers should be aware that, once again, the flexibility of the programme allows any similar coding-based approach to be employed should they be desired.

In the case of the CCM method, prospective researchers are able to rapidly open code the data in a line-by-line manner due to the structure of the data, with each post-it containing a single sentence, topic, or response to a survey question. These tags are user defined, and multiple tags may be applied to single notes allowing the rapid merging of codes during later coding stages. Subsequent grouping of open codes as axial-links and selective coding to highlight themes within the data could be conducted rapidly due to the visual nature of the environment clearly highlighting commonalities in the data and the ability to search for specific tags across the full dataset. A benefit of this process is the preservation of low-level data as the whiteboard may be viewed at any resolution from thematic to line-by-line.

However, as mentioned previously, if further analysis is required by prospective researchers the full dataset may be exported as a *.csv* file allowing for in-depth analysis using specialist software such as machine learning techniques or interaction with other research packages such as NVIVO.

### Case study—wearables for OAB management

To demonstrate the efficacy of the co-creation toolkit/recording approach, and provide prospective researchers with a step-by-step run-through of an example co-creation workshop, we conducted a case study that aimed to explore the unmet needs of people living with Overactive Bladder (OAB). The case study formed part of an ongoing research project aiming to assess the feasibility and potential design approach of a wearable device for the management of OAB. Here we report the outcomes of one of our co-creation workshops to report the strengths of the toolkit in practice.

#### Case study—WHY

As mentioned, the ideal co-creation process requires a clear understanding of the purpose behind the session (i.e., why stakeholder participation is necessary, what it brings to the project).

In this case, the first stage of the co-creation case study, like any other research project, was to build a thorough understanding of the research landscape. To do so a considerable literature review/market analysis was undertaken (see Case Study—HOW). This work provided the research team with a thorough understanding of the research and commercial landscape at the point of co-creation planning by: analysing the epidemiology of the condition (gathering a cross section of the target population), analysing the co-morbidities associated with the condition (giving an insight into the likely symptomatology of the cross-section), and analysing the present treatment techniques in place (allowing for an initial understanding of the likely experiences of this cross-section in regard to treatment/healthcare experiences).

This review was supplemented by expert discussion and informal conversation with stakeholders, which provided insight into the problem space and research needs. Given the wealth of literature available on the subject of treating OAB, the research team decided that at this stage informal expert discussion was sufficient, and that there was little need for any further “unknown-unknown” generating work. This will not be the case with every project however, as such prospective researchers should be open to the idea of conducting preliminary problem analysing research at this phase (e.g., open interviews or focus groups to define the research direction).

This initial exploratory work determined that the co-creation workshop would provide the greatest benefit if used to analyse the research problem from a *solutions-orientated perspective* (see Figure 1 WHY). Prospective researchers may come to different conclusions on the basis of this initial exploration of the research landscape. As such, the possibility of conducting additional exploratory research (e.g., literature reviews, pilot interviews, consultations with relevant experts) to fine-tune the co-creation to specific project goals should not be discounted.

#### Case study—WHAT

As mentioned, the second feature of the methodological framework should define the overall goal of the co-creation workshop. This is typically informed by the project rationale (WHY) but researchers should be aware that the nature of the stakeholder population and the timing of the workshop within the broader research project can alter this.

In the current case, the workshop was designed to address two outcomes, namely, actionable feedback on the possible design of the OAB management device, and additional insight into the condition to guide further co-creation workshops and research (see Figure 1 WHAT).

Given that members of the target population were willing and able to participate in a research project (this may not always be the case for every scientific question) it was not expected that the goals would need to change from this. Prospective researchers should be aware that information highlighted by the WHY phase, or specific population dynamics can change the research direction rapidly. Consequently, any planned co-creation should be amenable to change.

#### Case study—HOW

Several aspects of the HOW feature were chosen based on the goals and timing of the workshop within the broader research project. As mentioned above, at this point a significant literature review had been conducted to define the rationale for the co-creation (WHY) and the desired outcomes of the work (WHAT). However, prospective researchers should be aware that in many cases a review of the scientific literature alone cannot provide a complete understanding of the population’s needs.

Therefore, in this case, to fully contextualise the co-creation process, the pre-study literature review included a formal market analysis to provide the research team with an understanding of the commercial and medical landscape in addition to the scientific state of affairs. This literature review and market analysis was backed by a number of initial (informal) discussions with relevant experts within the field. These discussions included conversations with healthcare providers over the experiences of the population from the perspective of those administering treatment. This included conversations with nursing staff that would administer minor care, and specialist consultants to provide an insight into all levels of care. To provide additional insight, discussions were had with senior academic researchers, providing an understanding of the present state of the scientific landscape including its limitations, and critically unanswered questions. These conversations gave the research team invaluable guidance pertaining to the most relevant questions to ask to better inform the scientific understanding of the condition and target population. Finally, contact was made with relevant stakeholders in the commercial space, where discussions were had concerning the state of the medical device landscape. This provided the research team with an understanding of what devices were presently available (or under development), which supplemented the market analysis. Moreover, it provided critical insight into which prospective design directions would be feasible from a product design and manufacturing perspective—as well as giving researchers an idea of the approximate costs of any design process. This conversation framed the results of the co-creation workshop—allowing design features to be analysed and considered from a realistic perspective.

To gather actionable information on the specific feasibility and potential design of the device, several methods were selected. The primary data generation method was a workshop that facilitated conversation around a set of high-level topics informed by the contextualisation process. During the workshop, additional activities were conducted, including free-flowing discussions and surveys focused on the usability and appeal of example devices. Stakeholders also participated in an interactive prototype design task, where they built on earlier discussions to create their ideal device (see Figure 1 HOW).

During the workshop, one member of the research team served as a facilitator, playing a multifaceted role. Their primary responsibility was to ensure that everyone present could voice their opinions while also keeping the conversation focused and relevant to the topic. If stakeholders were hesitant to speak up, the facilitator prompted them to share their thoughts, ensuring their voices were heard. Additionally, the use of surveys provided a way for participants to express their opinions privately, free from any bias.

#### Case study—bias, tokenism, and power dynamics

Prospective researchers should at this point be keenly aware of potential sources of bias and how to avoid them at all stages of the co-creation process. Doing so is essential when conducting any form of inter- and transdisciplinary collaboration as contrasting perspectives must all be considered equally. Though the potential biases will differ based on the topic and goals of the co-creation, some common pitfalls should be considered. In particular, prospective researchers should ensure that any participant populations are free from tokenism. Participants should be empowered by the process, and engage in it willingly. This can be achieved by respecting participant perspectives, however varied, and considering their contribution as coming from core members of the research team rather than external advisors (37, 38). Wherever possible during the present case study, participants were engaged with as equals, with workshop facilitators making known that they were present to listen. Where conversation may have digressed beyond the scope of the workshop, rather than disagree or disregard this conversation this was considered additional insight.

In a similar vein, wherever possible potential researchers should consider how power-dynamics within participant populations may affect responses. For example, in the present case study the literature review highlighted a mistrust of medical professionals as a result of mistreatment. As such, it was determined that co-creation workshops should not mix the patient and healthcare provider populations at this phase, as doing so could influence how both parties acted and engaged with the workshop.

#### Case study—expert and stakeholder engagement

Prior to the formal conversational workshop, discussions were held with various relevant stakeholders. This included theoretical conversations with senior researchers from universities across Scotland, medical professionals, and individuals involved in the care sector to evaluate the current state of OAB management from both academic and medical perspectives. Results from the initial epidemiological and commercial literature search were condensed and discussed with these stakeholders to gather expert opinions. Though these discussions were guided by the literature data, rather than take a structured interview-like approach the discussions were informal in nature. This informal approach facilitated a frank and open conversation surrounding the present state of the field, its limitations and areas of interest (from an experienced perspective). Through understanding where experts believed the field was lacking, and where they believed the direction scientific research was heading, it was possible to create a list of topics that would be of considerable research value.

In addition to this expert insight, the research team also engaged with design firms to assess the practical considerations of a treatment device. These discussions focussed on assessing the feasibility of designing a wearable treatment device for the population from a commercial perspective.

This collaborative process facilitated the continual refinement of the co-creation framework, ensuring it was informed by all levels of technological readiness before being presented to the target population. In doing so, the subsequent analysis of the lived experiences of those suffering from OAB was grounded by a knowledge of present healthcare gaps, and an understanding of what features of the design would be reasonably achievable.

#### Case study—co-creation workshop

The formal conversational workshop contained conversational, interactive, and survey-based components. The overall flow of the workshop was guided by ten high-level topics, which included interactive components (see Table 1). These topics were designed to allow participants to first share their overall perspective and lived experience (providing contextualisation) before sharing specific considerations and partaking in interactive prototype evaluation and design.

**Table 1:** Case-study workshop conversational topics.

Section	Topic
1	Introductions
2	Life experience of OAB
3	Experience with treatment devices
4	Conversation around example device A
5	Conversation around example device B
6	Conversation around example device C
7	Conversation around example device D
8	Device rankings and why
9	Comments on prototype
10	Design ideas

Participants were recruited through the open advertisement of the workshop via standard channels (word of mouth, flyers, university newsletters/email databases etc.). The recruitment drive specified that prospective participants must be over the age of 18, and possess lived experience of Overactive Bladder or Incontinence in some form. Three participants that fit these criteria were recruited to take part in the workshop.

When recruiting for a co-creation workshop, prospective researchers should be aware of the number of available staff to assist with the research. Generally speaking, the sample size should not exceed the capabilities of the note-takers and facilitator. Though this will depend on the relative experience of the research staff, in this case it was decided that the sample size should not exceed $3\times N_{\mathrm{Notetakers}}$.

Three members of the research team attended the session, with one acting as a facilitator and the remaining two acting as note-takers (assigned 1, and 2 participants respectively). While this was within the acceptable limits of the workshop sample size (which had an upper limit of N=6 participants), it should be noted that the sample size was nonetheless limited. This is a critical point of consideration, as limited sample sizes will reduce the generalisability of any workshop findings. Therefore, if sample sizes are too small, or sufficiently large that all participants cannot be included in a single workshop, prospective researchers should be prepared to run multiple workshops to ensure robust results.

Participants were informed of the purpose of the workshop and the broad topics of discussion beforehand—however they were not made aware of the specific questions the research team were wishing to answer, to avoid bias.

Initial workshop discussion surrounded the lived experience of participants, exposure to previous treatment, and use of treatment devices in the past. Subsequent discussion centered around example devices, which were informed by the discovery phase and included key features likely to be important to those in this population.

Before discussing each device, participants filled in a brief survey, scoring their opinion of: the device’s design, if they would wear it, if they had heard of it before, and if they had used it before. After viewing all of the devices, participants were asked to rank them in order of preference at which point an open discussion was had as to why they had made this decision.

The final portion of the workshop involved participants discussing a prototype device, and designing their own ideal device taking into account all the conversation had up until this point. The prototyped device was only shown at the end of the session to ensure participants were not biased, and felt able to share their opinions freely. They were not aware there was a prototype device being worked on, nor that they would be shown it. This approach produced deep and considerable insight into the reasoning behind specific design considerations that would not have been possible with a traditional interview based approach.

#### Case study—qualitative analysis

The case study example described here utilised grounded theory to analyse the qualitative data generated during the workshop. Specifically, the Constant Comparative Method (CCM) was utilised. A CCM based analysis was chosen as it utilises a rigorous methodology to extract themes from a dataset with as little external bias from the reviewer as possible (39). This is particularly important in the field of healthcare research, where researcher bias surrounding may considerably sway the interpretation of stakeholder data (40, 41).

#### Case study—ethics statement

This research included live participants. All data obtained through any methodologies outlined in this publication were approved by the University of Edinburgh School of Informatics Research Ethics Committee (approval reference number: **7192**). Informed (written) consent was obtained from all participants prior to their participation in the study. Participants were fully informed of their rights in accordance with UK data protection law.

## Results

### Co-creation allows researchers to produce qualitative data rapidly and accurately

These results should demonstrate to prospective researchers the value of pre-workshop preparation. However, the results of this particular workshop should not be overstated. The example presented here serves as a guideline for prospective researchers rather than evidentiary research.

Additional work is required of any project wishing to undertake a co-creation workshop using this framework, as a larger sample size and further workshops will be required to produce scientifically robust results. Nevertheless, this framework demonstrates the contextualised and deep understanding of the problem space that may be produced by preparatory work, which is invaluable to conducting an effective co-creation. For example, the market analysis clearly highlighted the limitations of commercially available OAB treatment devices. These fell under the broad categories of battery life, construction/design, operation, and electrode design. Grievances with battery life (poor charge capacity, recharge times etc.), and operation (difficult instructions, simple interface) were as expected. In contrast, key insight was derived from the other categories, with users reporting poor opinion of hydrogel electrodes (“Runs out of stickiness after 3rd use.” “The pads supplied caused burns and irritated our skin but we ordered some for sensitive skin and they are fine”) and a desire for discreetness (“He likes the fact that he can use this product while at work. It’s convenient, discreet and it helps subdue or eliminate his discomfort.”). When combined with the epidemiological data obtained from the literature review these tools could show how the highlighted design limitations interacted with the population likely to suffer from OAB, manifesting as a set of personal unmet needs providing considerable problem insight. Guided by the toolkit, this stakeholder-informed approach allows for the formation of subsequent co-creation techniques deployed during the case study demonstrating the benefit of a prepared and informed approach to prospective researchers.

**Figure 4:**
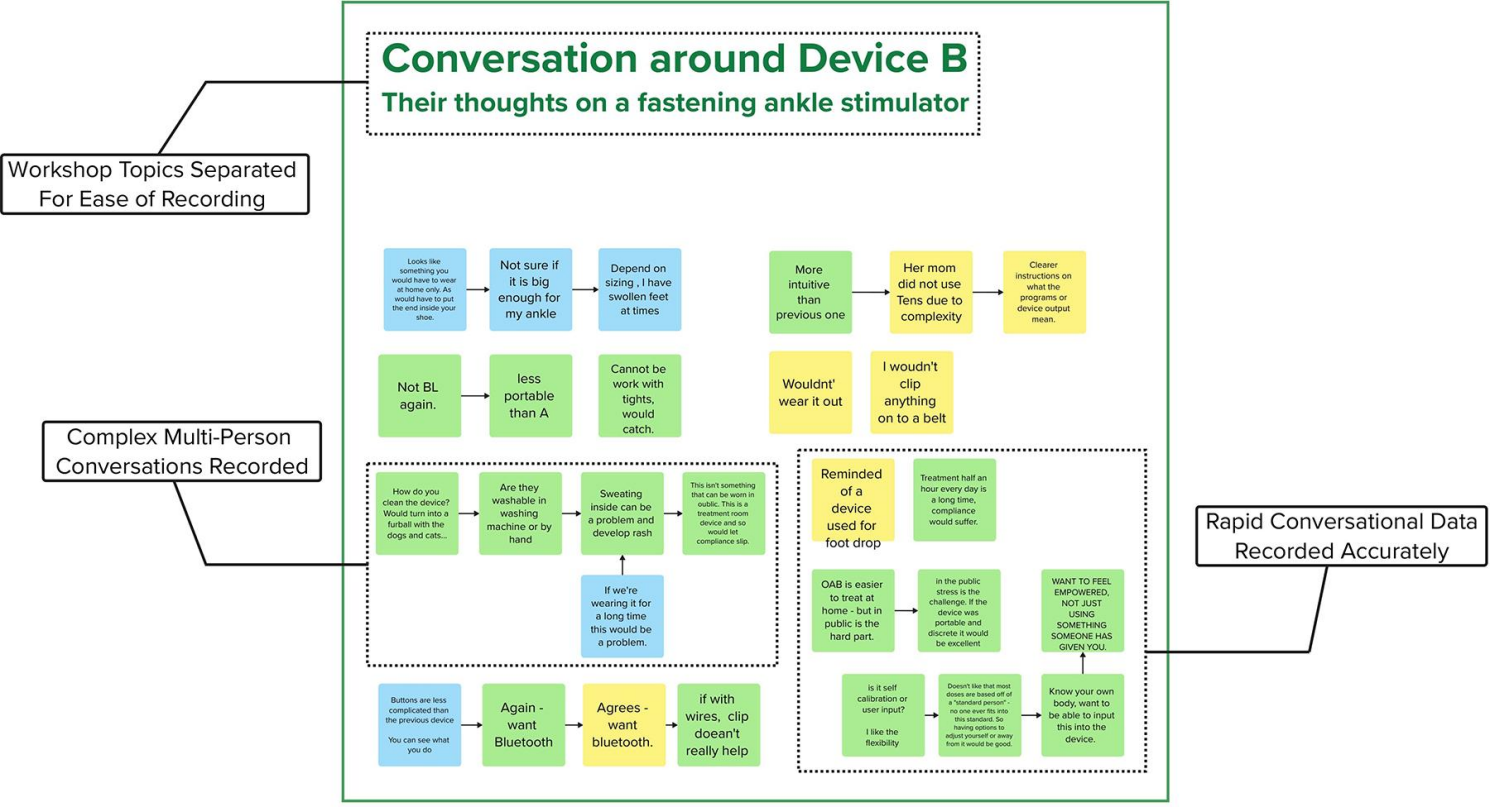
Example data from the case study workshop. Shown are lines of free-flowing conversation between three individuals. Arrows represent connected segments of conversation.

**Figure 5:**
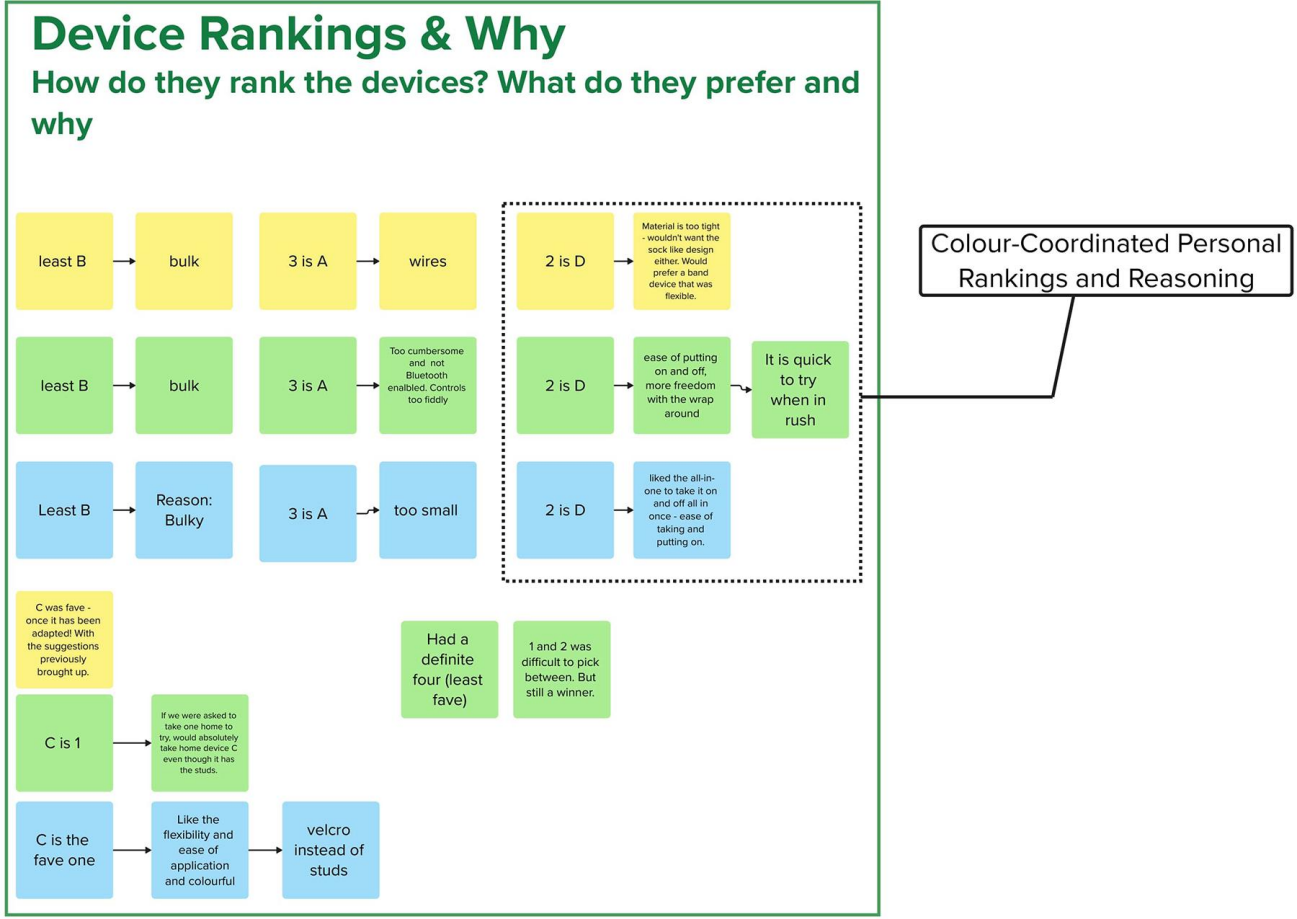
Example survey data from the case study workshop. Shown are each participant’s ranked opinion of the devices they were shown (4-Worst, 1-Best).

These results also demonstrate the benefits of using an electronic whiteboard to supplement traditional audiovisual recording technique. The color-coding used during the workshop allowed notetakers to rapidly distill both flowing conversation between several individuals (see Figure 4) and survey results (see Figure 5) in parallel without significant loss of information. This accuracy was seen when performing post-workshop error correction using an audio/video recording. There were very few occasions were notetakers failed to capture information (relying on the inclusion of a timestamp in it’s place) and the error correction did not highlight any serious errors in the data recorded. To this end, it is clear the technique exceeded expectations, allowing for the rapid and accurate recording of information that could be error-corrected far faster than a traditional line-by-line transcript through the use of timestamping. The use of multiple notetakers further saved time both during the workshop (increasing data recording speed) and afterwards (as mutual assistance further reduced the need for post-hoc error checking).

### Applying validated qualitative analyses generates robust co-creation outcomes

**Figure 6:**
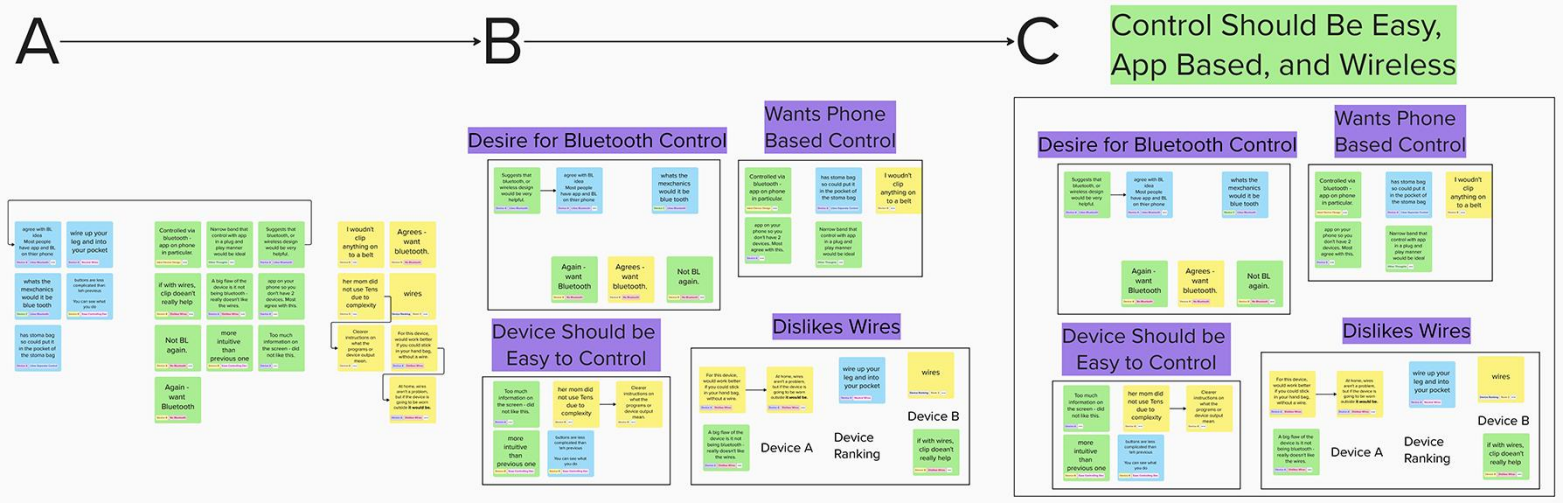
Example of the Constant Comparative Method (CCM) applied via the whiteboard environment. Method is split into three main coding stages: **(A)** Open coding, where each line of conversation is individually tagged. **(B)** Consolidation, where commonly tagged snippets are grouped (shown in purple). **(C)** Axial coding, where the code groups are analysed to determine broader axial codes that connect them (shown in green). The final stage of the CCM not shown is selective coding, where high-level axial codes are analysed to extract themes that connect them.

In addition to the quality of the raw data, the case study clearly demonstrates the efficacy of the approach detailed here in producing analysed results via validated qualitative analysis. Prospective researchers will see that the novel electronic environment allowed for rapid line-by-line open coding of the data (see Figure 6A). The tag-search function of the environment allowed these tagged lines to be compared, and where appropriate consolidated (combining common or overlapping tags, or editing them to be more accurate) with ease (see Figure 6B). This feature considerable accelerated the initial, potentially lengthy stage of the CCM (42) and facilitated the main feature of the method—comparison (43). Subsequent axial coding of the data was achieved through the use of this same search feature, where tagged objects could be rapidly grouped allowing for a clear visual consolidation of the dataset (see Figure 6C). Doing so made subsequent selective coding of these axial codes both faster, and more accurate as the visual nature of the environment allowed these groups to be rapidly assessed, consolidated, and analysed through the lens of connecting themes. The result of this analysis was a dataset that could be analysed at any level of organisation. At any point the researcher could “zoom in” on the datasets, observing individual tags or raw data to form in-depth ideas of potential themes that connected the data. Though this case study serves as a valuable starting point for prospective researchers, those wishing to conduct their own co-creation workshop should not be afraid to use other qualitative analytical techniques where required. Grounded theory serves as a very useful tool for bias-free analysis, however, the flexibility of the recording method described here could, in theory, allow for other techniques to be used if they prove more reliable (or if the research team is more experienced in these forms of analysis). Nevertheless, the results of the present case study analysis shall still be reported here for posterity and guidance for prospective research teams.

Applying the constant comparative method of analysis to the raw whiteboard database bore considerable information on participant design opinions. The online whiteboard environment allowed for conversational workshops, quantitative surveys, and interactive design activities to be analysed simultaneously. Doing so produced results that included specific design information and, critically, insight into the impacts of OAB.

The results were broadly split into a set of three high level “themes”: design features, materials preference, and contextualising OAB insight. In isolation, the first two themes provided valuable direction for the research project. The specific design preferences expressed by participants allowed for a solid foundation for further development. These included a band-like design:

“A strip that is not bulky at all. Would be flexible and length-ways along the device.”

with Bluetooth/App enabled control:

“A big flaw of the device is it not being Bluetooth—really doesn’t like the wires.”

“App on your phone so you don’t have 2 devices.”

Participants also expressed a desire for sweat-wicking comfortable material:

“Sweating inside can be a problem and [can] develop [a] rash.”

“Material should be soft.”

and rechargeable batteries:

“If it was a battery, once it ran out you’d need to get a replacement. This would mean they wouldn’t use it so much—or at all if they couldn’t make time to get a battery.”

The desire for these features was confirmed in the ideal device schematics proposed by the participants. As such, the approach was able to produce concrete design considerations of value to engineering and design teams.

While the device considerations obtained were undoubtedly useful to our ongoing OAB project, the central benefit of the toolkit to prospective researchers is the insight it provides. Analysing participant’s lived experiences of OAB provided critical additional information that explained the reasoning behind the specific design considerations noted. For example, the desire for non-invasive treatment options was likely explained by a history of invasive and unpleasant OAB treatments:

“[I] Had an operation in Summer 2015—pubic sling fitted. Caused other issues.”

“Just offered tablets and exercise—never anything else.”

In addition to providing deeper context, the insight explained seemingly paradoxical results surrounding a desire for discretion. Specifically, participants reported a desire for both flesh-colored and high-contrast designs:

“Could it come in a range of different skin tones in colors?”

“OR, can it be colorful?”

This mismatch was explained by a historical experience of stigmatisation as a result of OAB.

“Male consultant [said] it’s a woman’s problem”

*“Found the condition to be stigmatising.”*

Participants desired empowerment over their condition, seeking an opportunity to educate others about OAB through the use of a visible device (which would function as a conversation starter).

“if [it] bright people ask what it [is] for and can inform/educate [them] about OAB.”

These results clearly demonstrate the value of an insight-focussed approach. Prospective researchers should be aware of all of the ways in which their co-creation work may inform their understanding of a problem or research question. To use the present case-study as an example, a typical co-creation approach focusing solely on possible device designs would have failed to generate this insight, potentially producing conflicting results which could not be explained. As such, the insight-focussed nature of the framework described here produces actionable results. It should be remembered, however, that a real-wold project would require a larger sample size to produce scientifically robust results. Fortunately, this toolkit may be used to run additional workshops to build upon preliminary findings—in this case further examining prototypes possessing these features or conducting a deeper exploration of lived experiences.

As mentioned, prospective researchers should be aware that other forms of analysis may be more appropriate. As such, an additional analysis was conducted on a subset of the data to confirm that the CCM analysis performed on the electronic whiteboard was the most appropriate course of action for this particular study. The research team conducted the same CCM method on the dataset using NVIVO [an industry standard tool (44)]. It was found that the tools produced similar results. However, during the analytical process NVIVO represented these tags in a manner that was considerably more difficult to track, as a line-by-line transcript was the primary method of data presentation. This made the generation of thematic connections more time-consuming as there was little visual guidance to allow researchers to rapidly connect disparate ideas through common themes. As such, the research team was able to confirm that this was the best possible course of action without significant additional effort. Prospective researchers should always therefore be aware of the methods by which they can analyse their data, and which ones may be more appropriate. This may involve weighing up the benefits of an approach with which they are more experienced, vs. learning to apply a novel method which may be more appropriate. While there is no single answer to this question, conducting analyses on small subsets of the qualitative database can aid researchers in coming to a final decision.

### Co-creation can facilitate interdisciplinary collaboration

In addition to the previously discussed insight generated, the toolkit was found to offer a number of other benefits to prospective researchers.

The primary benefit of a collaborative co-creation endeavour such as the framework described here is the facilitation of interdisciplinary collaboration. In line with our predictions, by including relevant project engineers as notetakers during our case-study co-creation, we were able to improve the research efficiency of the ongoing project considerably. Prospective researchers should be aware that by directly exposing research and development staff at all levels of the translational pathway to the lived experiences of those living with OAB, they may facilitate in-depth discussions within a research team. By allowing these discussions to be had without the need for information transfer (e.g., via debriefings or formal meetings) the subsequent project research can progress significantly faster, and (in the case of the study described here) lead to the rapid iterative design of further prototypes within the scope of the unmet needs highlighted by the project. This method of recording allowed for the rapid circulation of anonymised data post-workshop. After completion of qualitative analysis the themes could be shared with researchers across disciplines in a manner that was universally accessible regardless of computer operating system setup. Where required, it was found that further analysis or integration with other program platforms was indeed possible via the use of data exporting in *.csv* format.

### Co-creation can assist with future research endeavours

A benefit of hosting co-creation events with relevant stakeholders that is not often reported within the literature is the ability to assist with other ongoing research projects. For example, the present case study workshop, which explored the lived experience of OAB, also revealed a considerable co-morbid overlap between OAB, under-active bladder, and urinary tract infection (UTI) within the attending population.

“Sometimes suffers overactive and under-active bladder—messages being disrupted due to MS”

“Has both OAB and UR (Urinary Retention), as well as UTI (Urinary Tract Infection).”

Although there is some evidence for an overlap between these conditions (45) it remains a little studied area of the literature. As such, by uncovering this link in the workshop the case study both contributed to ongoing research within the lab concerning OAB—and also led to further recruitment of individuals present at the workshop to other projects within the lab they were found to be appropriate for. In recruiting individuals through this channel, they were already comfortable with the lab staff and aware of the research environment. This allowed them to rapidly and seamlessly take part in other projects as participants. As such, prospective researchers should keep in mind the benefit a co-creation workshop can have for other projects that may be adjacent to the topic in question, as there may be an as of yet unknown link between them that may aid ongoing research.

## Discussion

This publication provides prospective researchers with a framework for conducting co-creation that has been thoroughly evaluated in a real world setting. Doing so highlighted the benefits and drawbacks of our approach, allowing prospective researchers to decide if the framework is appropriate for their use, and how it may be modified to best fit their specific research requirements.

A central benefit to the use of our combined electronic/audiovisual recording technique was the accuracy of the recorded results. The use of layered error correction techniques and instantly recognizable color-coding ensured note-takers were able to rapidly record both conversational and quantitative data. This accuracy is key when conducting large-scale workshops, which are relatively commonplace within the health and care sector (10) with several studies including upwards of thirty participants. In these situations, many parallel lines of conversation or data must be followed at once. Were traditional audio recording and transcription techniques used, considerable time would need to be spent to convert the data to a usable form [approximately three hours for every one hour of audio (46)]. In addition, allowing notetakers to support one another reduced fatigue further improving both the accuracy of the data recorded and increasing the theoretical possible length of planned workshops.

Further benefits to the moderator/notetaker support structure offered by our toolkit include the ability for the facilitator to focus completely on participants. By having the facilitator be a dedicated role with defined responsibilities, our toolkit falls in line with formal recommendations (47, 48) avoiding the problem of conversation being halted until the moderator has finished taking notes.

The accurate recording of conversational data allowed for the application of validated analytical techniques. The use of the electronic whiteboard environment allowed the constant comparative method to be rapidly carried out providing both actionable design data and vital insight into stakeholder’s lived experiences. This process was simple, with the initial open coding phase found to be easy using the in-built tagging system. The color-coded block system allowed granular data to be viewed rapidly even after final thematic analysis providing rapid access to key quotes or insight that backed up the emergent themes. This was particularly useful when analysing seemingly contradictory views (e.g., the desire for discreet, and non-discreet design options) which required both a high-level thematic understanding and a low-level grasp of stakeholder experiences. Importantly, these promising results were achieved at relatively little cost to researchers. The preparatory work using the toolkit and the recording of data via the electronic whiteboard environment did not require the use of expensive proprietary software. The premium version of the whiteboard cost approximately £18 per month (though a free version is also available), considerably less than established qualitative research tools such as NVIVO (the industry standard) which are priced at £95 per year for a student licence (increasing to upwards of £380 p/a for any other licence type) (49).

The limitations of the tagging system should not be ignored, however. The limit of 25 characters per tag, and 200 tags per dataset considerably constrained both the detail that could be captured during the coding process and the size of the dataset that could be analysed. While not an issue of the toolkit *per se*, it may change how the toolkit is used to conduct research. The toolkit may still be used to carry out large-scale co-creation endeavours, however where datasets are of sufficient size it may be required that researchers use industry standard tools suited for datasets of this type. This is not to say the novel whiteboard environment is without use however. Its ease of use and rapid turnaround times make the software well suited to a validation role, where researchers may independently analyse a section of the larger data to ensure inter-rater reliability (50) without significant time-loss.

Finally, the approach offered a number of additional benefits to researchers. Principally, the direct exposure of engineering staff to the lived experiences of stakeholders. Subsequent design analysis and conversation could be had considerably faster, as there was no need for information transfer between team members. Moreover, when working with raw design data, engineering staff were able to leverage a far deeper understanding of the problem space, leading to more effective, user-centered design decisions. Where individuals could not be directly present, the visual nature of the final data allowed the rapid circulation of anonymised results greatly increasing idea-sharing within the research environment.

Additionally, the insight gathered through the use of the toolkit highlighted key population parameters (i.e., the unexpected presence of an overlap between OAB and UTI) that directly contributed to ongoing research being conducted by the lab.

Using this co-creation framework, it was therefore possible to produce a robust context for co-creation study by analysing the unmet needs/problems faced by the population. Furthermore, when analysing this problem space the notetaking methodology’s layered error correction mechanisms and mutual assistance between multiple notetakers allowed for rapid, and importantly, accurate detailing of several classes of complex data. Knowing the accuracy of the recorded data is sufficient, it is possible to analyse how well the novel toolkit allows for the use of validated analysis.

Co-creation and Patient and Public Involvement and Engagement (PPIE) (51, 52) are often used interchangeably. However, the definition of PPIE falls short of our definition of co-creation. While PPIE provides considerable benefit for patients or other members of the public directly involved in the use of the service (53) it fails to address any other stakeholders that are a part of the whole system such as industrial manufacturing or management. However, if used correctly, the process of co-creation offers a number of benefits to all stakeholders from users to manufacturers (7). The primary benefit of the technique is the contextualisation of collected information (54). Co-creation based approaches allow researchers to effectively meet the needs of stakeholders by involving them in every stage of the research process, significantly improving treatment outcomes and stakeholder benefit (55).

### Limitations

Despite these clear benefits there were nevertheless several key issues with the approach that should be mentioned. The principle limitation of the approach was its limited applicability to larger datasets. First and foremost, as mentioned any workshop should not exceed the capabilities of the research staff. Too large a sample can lead to inadequate control by the facilitator, potentially introducing bias or preventing all participants from sharing their opinions. Alternatively, overloading attending notetakers can lead to gaps or mistakes in recorded data, requiring post-hoc error correction (e.g., manual correction of datasets from session recordings) which can require considerable time and effort.

In addition, the upper sample size of this methodology is constrained by the character and tag limits of the online environment, as well as poor browser performance when editing large whiteboards (or indeed, when many notetakers attempt to edit the same environment simultaneously) create an upper limit on the size of the projects that may be tackled using this technique. However, the performance of the toolkit and recording technique at a smaller scale cannot be denied.

In contrast, a particular limitation of this case study that prospective researchers should be aware of was an inadequate sample size. Though the impact of this issue was limited in this case, as the workshop findings were intended primarily as a guide for prospective researchers, in a real-world research project they must be considered. Inadequate sample size can lead to a biased set of themes, which generalise poorly. At the outset of a research project, prospective researchers should allocate the time and resources to potentially conducting multiple workshops if required. The findings of these workshops should be comparing to ensure that any discovered themes are valid.

Determination of what constitutes an adequate sample size can be a difficult task in co-creation. As the research is qualitative, a standard power analysis is not possible. Therefore, if utilising the constant comparative method (CCM) as described above researchers should instead aim to reach a point where no new themes emerge from the data (known as thematic saturation) (56) at which point it is generally considered that the sample size is sufficient for the given methodology (i.e., no further information can be extracted). Given that the sample size of the case study was limited, and only a single workshop was conducted this cannot be said to have occurred. Therefore, prospective researchers should conduct additional analyses or workshops to check that a sufficient sample size has been achieved. Though no single answer to this question exists, prior research indicates that as a rule of thumb, 9–17 interviews or 4–8 focus group discussions are typically required to reach saturation (57).

Further limitations were seen in the application of surveys as part of the case study. It was found that while they could be rapidly administered and recorded using the online environment, the results obtained were of little use when compared to the conversational and interactive design components of the workshop. This limitation may have been due to the nature of the workshop goals however, (the “WHAT” feature) which were better addressed with a more open approach. To this end, future work could instead apply survey-based data collection at a different stage (WHY) in the research project.

## Conclusion

We presented a framework that demonstrates the ability to conduct co-creation in a low-cost manner, producing information with considerable breadth, and depth, through timely analysis. The visual nature of the data recording technique allows for rapid dissemination of key design considerations and, more importantly, critical insights. While there are some limitations in handling large datasets, the toolkit nonetheless serves as a solid foundation for developing future co-creation methodologies. As such, prospective researchers should consider the limitations of the approach described here when planning their own co-creation endeavour. Moreover, they should not be afraid to modify and adjust the framework where needed, all co-creation projects are unique and require a tailored approach to maximise their usefulness.

Although this paper focused on a single case study to demonstrate the effectiveness of the approach, and provide prospective researchers with a detailed account of a typical co-creation event, the research team has subsequently applied our approach to multiple co-creation projects within the digital health and care space. These projects, which included alert services for home use, UTI detection, and overdose monitoring, required customised co-creation approaches developed through this flexible, modular approach. We hope that this work will serve as a valuable starting point for prospective researchers wishing to begin, or continue, their journey in qualitative healthcare research from an engineering background.

## Data Availability

The raw data supporting the conclusions of this article will be made available by the authors, without undue reservation.
